# Effects of ageism on burnout among clinical nurses

**DOI:** 10.1371/journal.pone.0313043

**Published:** 2024-11-07

**Authors:** Sukjae Park, Hyunmin Lee, Minsook Seo, Hee Kyung Kim, Eunhee Shin

**Affiliations:** 1 Department of Nursing, Kangbuk Samsung Medical Center, Seoul, Republic of Korea; 2 Department of Nursing, Samsan Hospital, Wonju, Republic of Korea; 3 Department of Nursing Science, Sangji University College of Health Sciences, Wonju, Republic of Korea; Cardiff University, UNITED KINGDOM OF GREAT BRITAIN AND NORTHERN IRELAND

## Abstract

Nurses, as healthcare professionals who frequently interact with older patients in clinical settings, play a crucial role in providing direct care and services to older patients. However, nurses caring for older patients often experience substantial physical and emotional exhaustion, which can trigger a loss of motivation, feelings of helplessness, and burnout. The current study, conducted in Korea, investigated the impact of ageism on burnout among clinical nurses. To determine whether ageism was a significant predictor of nurse burnout, a structured questionnaire was distributed to 332 nurses at two hospitals. Multiple regression analysis then showed that ageism (B = .066, p < .001), female sex (B = .530, p = .003), and lower preference for geriatric nursing (B = .357, p = .002) were significantly associated with burnout. Our findings confirmed that ageism was strongly associated with nurse burnout, suggesting the need to develop interventions or training programs that can change nurses’ perception of older people and facilitate emotional change.

## Introduction

Owing to the unprecedented advancements in science, technology, and medicine, human life expectancy has continued to steadily increase. In particular, South Korea stands as one of the leading nations in terms of healthcare, with an average life expectancy reaching a 83.3 years and surpassing the national averages observed in Organization for Economic Cooperation and Development (OECD) countries (i.e., 81.0 years), positioning it among the top-ranking nations within the OECD [1]. This heightened life expectancy mirrors the rapid aging of the South Korean population, with predictions estimating that the older population will constitute 24.3% of the total population by 2030, increasing further to 40.1% by 2060 [2].

The rapid growth of the older population in South Korea has escalated social costs in various areas, such as pensions, healthcare, and welfare [3], amplifying intergenerational distribution issues considering the limited socioeconomic resources [4]. The widening gap in generational values and attitudes, coupled with the spread of ageism in this societal atmosphere, has positioned older people as subjects of negative perceptions and prejudices, characterized by the view that they are outdated and primarily in need of care [3]. Hence, seniors in present-day South Korea face age discrimination, otherwise known as ageism, on par with the gender, age, health status, and socioeconomic discrimination observed in advanced nations [5]. The increase in the older population and the high prevalence of chronic diseases in South Korea require long-term treatment and nursing, so the demand for nursing services for older patients in clinical settings is inevitable [6].

Ageism, first defined by Butler [7] in the late 1960s, is a form of prejudice and discrimination directed toward individuals based on their age, similar to how sexism and racism discriminate against people based on their biological sex or skin color. Iversen [8] defines ageism as a negative or positive stereotype, prejudice, or discrimination against older people based on their chronological age. Ageism can be categorized into cognitive aspects (stereotypes), affective dimensions (prejudice), and behavioral components (discrimination). Moreover, ageism can be divided into individual (micro-level), social (meso-level), and institutional or cultural levels (macro-level), indicating that societal and cultural backgrounds or perceptions can influence ageism beyond an individual’s ageist beliefs or feelings. Studies have shown that ageism can have a detrimental impact on the mental and physical well-being of older individuals, including increased feelings of depression, loneliness, chronic health conditions, and a decline in subjective health status [9, 10]. Consequently, proactive efforts that address and reduce ageism are urgently needed.

As healthcare professionals who frequently interact with older individuals in clinical settings, nurses play a crucial role in providing direct care and services to older people [6]. With the increasing utilization of healthcare services by older adults, nurses are becoming key agents in addressing health-related issues and promoting the well-being and quality of life among older people [11]. However, caring for older patients can be demanding given that nurses need to pay special attention to the risk of falls, engage in frequent monitoring to prevent adverse events, and provide repeated explanations, which can cause physical and emotional fatigue. Hence, several studies have reported that nurses and student nurses exhibit unfavorable attitudes toward older people [12–15]. In particular, research has shown that nursing students had more negative attitudes toward older people than qualified nurses [15] and that ageism exhibited by nurses had a considerable influence on the dignity and autonomy of older patients [16]. Consequently, nurses caring for older people often experience high levels of physical and emotional exhaustion, which can trigger a loss of motivation, feelings of helplessness, and burnout [17, 18].

The most widely known theoretical definitions of burnout in people whose jobs involve helping others or dealing with clients [19] are emotional exhaustion, which reflects the depletion of psychological resources to interact with those they help, depersonalization, which is a cynical attitude toward patients, and a reduction of efficacy and competence in the work role, which is a feeling of lack of personal accomplishment. In organizations, burnout can be conceptualized as the opposite of work engagement, which involves members using their cognitive, physical, and emotional selves [19], and among these, the emotional exhaustion aspect can be said to be the core of burnout. In reaction to emotional exhaustion, individuals experience a reduced personal accomplishment and depersonalization or cynicism.

Nursing is a stressful job that exposes to prolonged stress and stressors. This may be because they are exposed to a variety of excessive demands, such as staff shortages, poor resource availability, low pay, awareness of working too many hours, shift work, role conflict, lack of sleep, overtime, as well as overloading of clients or patients. The frequency of particular stressors may vary across professions, but perception of a stressful work life may be a common factor [20]. In addition, there may be differences in some aspects of burnout that stress can predict, and other aspects of stress can also influence burnout differently. Emotional dissonance has been proposed as one possible explanation for the relationship between ageism, stress, and burnout [21]. This occurs in situations where one has to express an emotion different from what a professionals feels based on organizational or cultural expectations [22]. For nurses, it may come from a requirement to suppress feelings that are deemed inappropriate or potentially offensive while showing culturally preferred emotions [23]. This discrepancy of emotions allows nurses to be placed in a state of emotional dissonance with respect to ageist attitudes. Nurse burnout is a negative work stress response that occurs when nurses fail to cope with the repetitive or continuous stressors they encounter in their workplace [24]. Nurses experiencing burnout may compromise their own health, experience job dissatisfaction, and adopt negative professional attitudes, which can potentially undermine the quality of patient care [25]. Moreover, burnout often impacts not only the affected individuals but also their colleagues other healthcare personnel, and even patients [26]. In line with this, studies have shown that a positive relationship exists between ageism and burnout of nurses working in hospitals and that negative perceptions of older people and ageism can increase burnout by lowering the level of emotional exhaustion, depersonalization, and personal achievement of nurses [26]. Therefore, to reduce burnout among hospital nurses caring for older people and enhance the quality of geriatric nursing care, it is essential to first assess and address the extent of ageism among nurses, which can influence burnout.

Only a limited number of both domestic and international studies have been investigated the relationship between ageism and nurse burnout. In South Korea, for instance, prior studies on ageism have predominantly focused on specific concepts, such as contact with older people, attitudes, preference for geriatric nursing, levels of education about aging, geriatric anxiety, knowledge about aging, the frequency and quality of interactions with older individuals, and age-discriminatory behaviors [27, 28]. A recent study conducted in Israel reported a positive correlation between nurse burnout and ageism among nurses working in long-term care facilities [21]. However, research on the relationship ageism and burnout in nurses is very limited. Hence, the current study aimed to investigate the impact of ageism on burnout among ward nurses based on the assumption that higher levels of ageism may be associated with increased levels of burnout. This research proposes the following hypothesis whether ageism could be a significant predictor of nurse burnout.

## Materials and methods

### Study design

We utilized a cross-sectional study design to determine whether ageism among nurses caring for older patients was a significant predictor of burnout.

### Study subjects

Our participants included nurses who had worked for over 6 months in a comprehensive hospital with over 250 beds and had experience in caring for older patients aged 65 or older in wards other than pediatrics, surgery, and neonatal care. These nurses also understood the necessity and purpose of the study and confirmed their participation in writing after comprehensively understanding the study’s aims. Using the G*Power program version 3.1.9 [29], the required number of participants was calculated to be 263, considering 0.05 type I error, 0.99 power, 0.15 effect size, and 16 predictor variables in the linear multiple regression analysis. Therefore, we planned to enroll 368 participants, considering a 25% dropout rate. Recruitment was conducted until the required sample size was attained along with some cases of refusal to participate. Finally, data for 332 participants were used for statistical analysis after excluding 36 participants with incomplete answers.

### Research tools

The structured questionnaire used in this study contained a total of 55 questions: 15 on general characteristics, 18 on ageism, and 22 on burnout.

### General characteristics

The characteristics of the participants included gender, age, education, marital status, religion, clinical experience, employing institution, department, and residency while growing up. We also determined whether participants had experience dealing with older people, which included whether they had received education in geriatric nursing, their past experience of living with older people, their current living arrangements with older people, their experience in older people volunteer activities, geriatric anxiety, and their preference for geriatric nursing.

### Ageism

Ageism was measured using the Korean version [30] of the Fraboni Scale of Ageism (FSA) developed by Fraboni, Saltstone, and Hughes [28]. This tool contains 29 questions about ageism, each of which is scored using the following scale: 1 (strongly disagree), 2 (disagree), 3 (agree), or 4 (strongly agree). The total FSA score is the sum of scores for all 29 questions, with higher scores indicating greater ageism. To validate the Korean version of the FSA, Kim, Min and Kim [30] conducted exploratory factor analysis and confirmed a three-factor structure consisting of affective avoidance, discrimination, and stereotyping. This study used the same three-factor structure and total FSA score. The Cronbach’s α was 0.86 at development [28] and 0.82 in the Korean version [30]. This study showed a Cronbach’s α value of 0.90, with the emotional avoidance, discrimination, and stereotyping having Cronbach’s α values of 0.86, 0.79, and 0.79, respectively.

### Burnout

Burnout was examined using the Maslach Burnout Inventory (MBI) [31], translated into Korean and validated by Choi [32]. This tool comprises a total of 22 items under 3subdomains: emotional exhaustion (9 items), depersonalization (5 items), and decreased personal accomplishment (8 items). Each question is evaluated on a 7-point scale (0–6 points). The MBI is a highly reliable and valid tool for assessing burnout in personal service occupational groups, including nurses [31]. Choi’s [32] validation study showed that the MBI has a Cronbach’s α of 0.76. This study showed a Cronbach’s α value of 0.93, with the emotional exhaustion, decreased sense of self-accomplishment, and subdomains having a Cronbach’s α value of 0.90, 0.89, and 0.85, respectively.

### Ethical considerations and data collection

To ensure ethical consideration of the subjects before conducting study, approval from the Institutional Review Board (IRB) of the institutions to which the study subjects belonged was received after review, and the study was performed after explaining the purpose of the study and asking for cooperation from the hospital’s nursing headquarters. The researchers provided a written document explaining the purpose of the study, voluntary participation, and anonymity, as well as a consent form for nurses who wished to voluntarily participate in the study via a recruitment notice. Those who voluntarily consented to participate in the study signed the consent form and were requested to complete the survey.

After obtaining approval from the KBSMC IRB No. 2023-07-016-002), structured self-administered questionnaires were used to collect data between August 1, 2023 and October 30, 2023. The researchers visited the hospitals and posted announcements for study participation. The questionnaires were distributed to and collected from the nurses who agreed to participate. The participating nurses were briefed about the study objectives, confidentiality of data, anonymity, and freedom to refuse or cease participation at will. The questionnaire was completed only once. Nurses spent approximately 30 min listening to the study objectives and completing the questionnaire. Participating nurses were provided coupons for drinks.

### Data analysis

Data were analyzed using SPSS 26.0 (IBM Corp., Armonk, NY, Unites States). Participants’ general characteristics, characteristics related to older people, burnout, and ageism were analyzed using frequencies, percentages, means, and standard deviations. Differences in burnout and ageism according to the participants’ characteristics were analyzed using t-tests and one-way analysis of variance. Correlations between participants’ burnout and ageism were analyzed using the Pearson correlation coefficient. Associations between participants’ characteristic, burnout, and ageism were analyzed using multiple regression analysis.

## Results

### General and geriatrics-related characteristics

Table 1 summarizes the general characteristics of the subjects. Among the included participants (average age, 29.59 years), 94.6% were female, 81.9% had completed a four-year university course, 74.1% were unmarried, and 66.6% reported having no religious affiliation. The average medical facility tenure of the participants was 5.5 years, with those having a tenure of 5 years or more being most prevalent at 42.5%, followed by those with a tenure of over 1 to 3 years at 30.1%and then those with a tenure of under 6 months to <1 year at 8.7%. The majority of the participants (90.1%) worked in tertiary hospitals, whereas 9.9% worked in hospitals or general hospitals. The department distribution of the participants was as follows: internal medical wards (42.2%), surgical wards (34.3%), intensive care units (13.0%), emergency rooms (8.4%), and other departments (2.1%). The majority of included participants (58.1%) were brought up in urban areas.

**Table 1:** General and geriatrics-related characteristics of subjects (N = 332).

Variables	Categories	n (%) or mean±SD
Gender	Male	18(5.4)
	Female	314(94.6)
Age (yr)		29.59±5.62
	≤29	201(60.5)
	30~49	126(38.0)
	≥50	5(1.5)
Level of education	Diploma	29(8.7)
	Bachelor’s degree	272(81.9)
	Above Master’s degree	31(9.3)
Marital status	Single	246(74.1)
	Married	86(25.9)
Religion	Yes	111(33.4)
	No	221(66.6)
Work experience (yr)		5.5±63.56
	0.6~<1	29(8.7)
	≤1~<3	100(30.1)
	≤3~<5	62(18.7)
	≤5	141(42.5)
Current department	Internal ward	140(42.2)
	Surgical ward	114(34.3)
	ICU	43(13.0)
	ER	28(8.4)
	Others	7(2.1)
Area of residency while growing up	Metropolitan city	193(58.1)
	Small city	109(32.8)
	Countryside	30(9.0)
Geriatric education	Yes	257(77.4)
	No	75(22.6)
Residential experience with elders	Yes	165(49.7)
	No	167(50.3)
Residential status with elders	Yes	24(7.2)
	No	308(92.8)
Volunteer experience with elders	Yes	262(78.9)
	No	70(21.1)
Aging anxiety	Yes	205(61.7)
	No	127(38.3)
Preference for geriatric nursing	Yes	73(22.0)
	No matter	183(55.1)
	No	76(22.9)

Characteristics of the subjects’ experience in dealing with older people are summarized in Table 1. Accordingly, 77.4% of the respondents reported having experience with older people-related education, whereas 22.6% reported no such experience. Moreover, 49.7% of the respondents responded affirmatively to having past experiences residing with older people, whereas only 7.2% reported currently living with older people. Furthermore, 78.9% reported having experience with volunteer activities related to older people, whereas 61.7% responded affirmatively to having anxiety about aging. In terms of preference for geriatric nursing, 22.0%, 55.1%, and 22.9% responded “preferred,”“neutral,”and “not preferred.”

### Ageism and burnout

The extent of ageism and burnout among the included subjects is detailed in Table 2. Accordingly, our results showed that participants had an average score of 2.31±0.46 for ageism and 2.92±0.58 for burnout. Among the subdomains of ageism, emotional avoidance scored highest at 2.54, followed by discrimination at 1.91±0.55 and stereotypes at 2.42±0.52. Within the subdomains of burnout, emotional exhaustion scored highest at 3.57±1.14, followed by reduced personal accomplishment at 2.11±1.05 and depersonalization at 3.06±1.38.

**Table 2:** The extent of geriatric ageism and burnout among the subjects (N = 332).

Variables	Range	M±SD	Minimum	Maximum	Skew	Kurtosis
**Ageism**	0–4	2.31±.46	1.39	4.00	1.422	2.920
Emotional avoidance	0–4	2.54±.55	1.00	4.00	.575	.646
Discrimination	0–4	1.91±.55	1.00	4.00	1.413	2.874
Stereotyping	0–4	2.42±.52	1.00	4.00	.697	1.350
**Burnout**	0–6	2.92±.58	.55	5.82	.579	1.057
Emotional exhaustion	0–6	3.57±.1.14	.22	6.00	−.149	−.241
Personal accomplishment	1–6	2.11±.1.05	.00	5.75	1.089	1.771
Depersonalization	1–6	3.06±.1.38	.00	6.00	−.087	−.574

### Differences in ageism and burnout according to general and geriatrics-related characteristics

Differences in ageism and burnout based on general characteristics are outlined in Table 3. Notably, ageism was higher among respondents between the ages of 30 and 49 years than among those under 29 years (F = 4.712, *p* = .101). Furthermore, married respondents showed higher levels of ageism than did unmarried respondents (t = −3.263, *p* = .001), whereas those who reported having a religious affiliation exhibited higher ageism than did those who reported having no religious affiliation (t = 2.018, *p* = .045). Moreover, respondents with a medical institution tenure of over 5 years showed higher levels than did those who had a tenure of 1–<3 years (F = 9.709, *p* < .001). We found that ageism was higher among those working in internal medicine wards than among those working in surgical wards (F = 4.861, *p* = .001). Regarding burnout, females demonstrated higher burnout levels than did males (t = −2.307, *p* = .022).

**Table 3:** Differences in geriatric ageism and burnout according to general characteristics (N = 332).

Variables	Categories	Ageism		Burnout	
		M±SD	t/F(*p*)	M±SD	t/F(*p*)
Gender	Male	2.30±.49	−.037(.970)	2.42±.1.00	−2.307 (< .022)
	Female	2.31±.45		2.95 ±.94	
Age (yr)	≤ 29 ^a^	2.25±.41	4.712(.010) a < b	2.92±.88	.660(.518)
	30~49 ^b^	2.40±.50		2.93±.1.06	
	≥ 50	2.33±.13		2.44±.62	
Level of education	Diploma	2.38±.52	1.783(.170)	2.97±.95	1.362(.258)
	Bachelor	2.28±.45		2.94±.94	
	Above Master	2.42±.43		2.65 ± 1.06	
Marital status	Single	2.26±.42	−3.263(.001)	2.95±.87	.930(.350)
	Married	2.44±.42		2.82 ± 1.15	
Religion	Yes	2.39±.58	2.018(.045)	3.03 ± 1.12	1.143(.155)
	No	2.27±.38		2.87±.84	
Work experience (yr)	0.6~ <1	2.29±.40	9.709 (< .001) a < b	2.98±.79	.158(.925)
	≤1~ <3 ^a^	2.13±.28		2.87±.71	
	≤3 ~ <5	2.31±.46		2.95±.98	
	≤5 ^b^	2.44±.46		2.93±.1.11	
Current department	Internal ward ^a^	2.42±.54	4.861(.001) a>b	3.03±.1.11	1.506(.200)
	Surgical ward ^b^	2.20±.36		2.87±.77	
	ICU	2.32±.42		2.91±.82	
	ER	2.18±.30		2.70±.91	
	Others	2.11±.22		2.38±.67	
Area of residency while growing up	Metropolitan city	2.27±.40	2.697(.069)	2.87±.87	1.889(.153)
	Small city	2.33±.47		2.92±.99	
	Countryside	2.46±.46		3.23 ± 1.21	
Geriatric education	Yes	2.30±.47	−.309(.757)	2.91±.97	–.516(.606)
	No	2.32±.42		2.97±.87	
Residential experience with elders	Yes	2.32±.52	.507(.612)	2.95±.1.06	.630(.527)
	No	2.29±.38		2.88±.83	
Residential status with elders	Yes	2.29±.57	−.164(.870)	2.84±.1.24	−.423(.672)
	No	2.30±.45		2.92±.92	
Volunteer experience with elders	Yes	2.28±.44	−2.059(.040)	2.91±.91	−.363(.717)
	No	2.41±.51		2.95 ± 1.09	
Aging anxiety	Yes	2.36±.51	2.859(.005)	3.06 ± 1.03	3.612 (< .001)
	No	2.22±.33		2.70±.75	
Preference for geriatric nursing	Yes ^a^	2.12±.33	63.587 (< .001) a, b <c	2.57±.79	39.727 (< .001) a,b < c
	No matter ^b^	2.20±.28		2.74±.73	
	No ^c^	2.74±.46^c^		3.38±.1.15	

Differences in ageism and burnout based on older people-related characteristics are depicted in Table 5. Ageism varied based on experience with older people-related volunteer activities such that respondents who reported having no such experience exhibited higher levels of ageism than did those who had relevant experience (t = −2.059, *p* = .040). Higher levels of ageism were observed among respondents with anxiety about aging than among those who did not (t = 2.859, *p* = .005). Concerning preferences in geriatric nursing, higher ageism was noted among those who reported not preferring geriatric nursing than among those who expressed preference or neutrality (F = 63.587, *p* < .001). Based on older people-related characteristics, higher levels of burnout were observed among those with anxiety about aging than among those without the same (t = 3.612, *p* < .001). Similarly, regarding preference for geriatric nursing, higher levels of burnout were noted among respondents who reported not preferring geriatric nursing than did those who expressed preference or neutrality (F = 39.727, *p* < .001).

### Correlation coefficient between ageism and burnout

Table 4 shows that ageism and each dimension of ageism were significantly correlated with burnout and each of dimension of burnout. This suggests that higher levels of ageism were significantly correlated with higher levels of burnout.

**Table 4:** Correlation coefficient between ageism and burnout (N = 332).

변수		Ageism	EA	DI	ST	Burnout	EE	PA	DE
**Ageism**		1							
EA			1						
DI			.603**	1					
ST			.502**	.623**	1				
**Burnout**		.607**				1			
EE			.345**	.353**	.336**		1		
PA			.493**	.586**	.422**		.359**	1	
DE			.421**	.412**	.395**		.709**	.413**	1

*p < .01

** EA, emotional avoidance;DI, discrimination; ST, stereotyping

*** EE, emotional exhaustion, PA, personal accomplishment, DE, depersonalization

### Factors influencing burnout

Before performing regression analysis, autocorrelation of dependent variables and multicollinearity between independent variables were reviewed. The Durbin–Watson index was used for autocorrelation, with our results showing a value of 1.771, which is within the standard value of 1–3. This confirmed that the variables were independent without autocorrelation. Tolerance and variance inflation factor (VIF) were used to determine multicollinearity between independent variables. The tolerance limit was found to be 0.720–0.998, which is more than 0.1, whereas VIF was found to be1.030–1.390, which is less than 10, indicating that multicollinearity was possible, no problem existed, and that our data was suitable for regression analysis. Accordingly, multiple regression using all selection methods found that ageism (B = .1.066, p < .001), gender (B = .530, p = .003), and preference for geriatric nursing (B = .357, p = .002) significantly affected burnout such that respondents with higher levels of ageism, female respondents, and those who showed no preference for geriatric nursing demonstrated higher burnout. The explanatory power of these variables for burnout was40.4%. Ageism (β = .521) was found to have the greatest impact on burnout, followed by preference for geriatric nursing (β = .158) and gender (β = .082) (Table 5).

**Table 5:** Factors influencing burnout among clinical nurses (N = 332).

Variables	B	β	t	*P*	F(*p*)	AdjR^2^
(constant)	−.220				57.017 (< .001)	.404
Ageism	1.066	.512	10.259	< .001		
Gender (F = 1, M = 0)	.530	.127	2.985	.003		
Aging anxiety (Y = 1, N = 0)	.160	.082	1.909	.057		
Preference for geriatric nursing (No = 1 Yes = 0)	.357	.158	3.163	.002		

## Discussion

The current study was conducted to determine the level of burnout and ageism among clinical nurses and clarify how ageism affects nurse burnout. Notably, our results showed that the respondents had an average ageism score of 2.31 out of 4, which was similar to the ageism score of 2.30 points for nurses working in medical surgical units in Indonesia [33] and those working at domestic tertiary general hospitals [34]. Interestingly, our ageism score was higher than that reported in a study conducted on nurses at a general hospital (2.21 points) [10] but was lower than that presented in a study conducted on nurses at another general hospital in Korea (2.63 points) [35]. As such, apparent differences exist between our scores for the level of ageism among nurses and those reported in previous studies both domestic and international, although such differences were found to be moderate, as revealed in previous studies [34, 35]. This can be explained by the findings of a previous study [27], which revealed that doctors had the highest ageism score, followed by nurses and other related professionals among medical professionals, and nurses maintain a relatively neutral attitude toward older patients while recognizing them as care recipients. This is reported to be due to the fact that nurses have recently increased their tendency to recognize older patients as neutral care subjects based on undergraduate education and clinical practice experience rather than treating older patients due to factors such as personal nursing preferences, contact experiences, and aging anxiety [27].

The current study found that nurses had an average burnout score of 2.92 out of six. This is consistent with the results of previous studies conducted at a domestic general hospital, which showed that nurses in the nursing care integrated service ward had an average burnout score of 3.00 points [36], those caring for chronically ill patients exhibited an average score of 2.96 points [37], and those caring for older patients, a demographic similar to that studied herein, displayed an average score of 2.91 points [38].

Our findings showed that nurse burnout was positively correlated with ageism, with respondents having higher burnout scores demonstrating greater stereotypes, prejudice, and discriminatory behavior toward older patients. This was consistent with the results of a study on nurses at long-term care facilities in Israel, which reported that those with higher ageism scores exhibited greater burnout [21]. In addition, studies on the relationship between ageism and burnout have been conducted in diverse healthcare professionals [19], and higher overall burnout, emotional exhaustion, and depersonalization are associated with ageism, and those with ageist attitudes are also reported to have low level of personal accomplishment. Given the lack of prior research on the relationship between nurse ageism and burnout, direct comparisons of the relationship between the two variables remains difficult. However, one study showed that burnout caused by continuous and repetitive emotional pressure can trigger negative attitudes toward others [39]. It has been reported that there is a strong correlation between emotional dissonance and emotional exhaustion and cynicism [23]. It indicates that emotional dissonance puts nurses in a state of tension that strongly predicts burnout. This can be explained by the fact that emotional dissonance represents a threat to an individual’s identity, reducing the likelihood of voluntary emotional expression and requiring more regulatory strategies [22]. This in turn further depletes the emotional resources of nurses and leads to more burnout. Burnout is a common consequence in clinical situations where nurses face long-term stress or excessive demands, and such situations can contribute to emotional exhaustion and depersonalization. In addition, nurses are placed in stressful situations as they experience emotional dissonance resulting from a requirement to suppress emotions that are deemed inappropriate or potentially offensive while showing culturally preferred emotions, which affects burnout in relation to ageist attitudes [22, 23].

The results of our analysis showed that factors affecting nurse burnout included ageism, preference for geriatric nursing, and gender such that respondents with higher ageism scores, those with lower preference for geriatric nursing, and female respondents demonstrated higher burnout. These variables showed an explanatory power of around 40.4%. Among these, ageism appeared to have the greatest impact on nurse burnout. Considering the little prior research on ageism as a factor affecting nurse burnout, direct comparison remains difficult; nonetheless, we will attempt to discuss the context of each variable. Nurses are professionals who have the most direct and continuous contact with patients in hospitals and experience higher work-related stress and burnout than do individuals with other occupations [40, 41]. In particular, studies have shown that caring for critically ill patients frequently leads to burnout [38] and that older patients have diverse and complex health problems that demand a workload greater than that required for other age groups [11].

The high workload and increased psychological demand experienced by nurses have been reported to cause burnout [42], which increases the emotional exhaustion of nurses [31]. In fact, among the subdomains of burnout analyzed in the current study, emotional exhaustion showed the highest score, which can lead to the development of negative emotions and attitudes toward others [31]. At the same time, it was shown that personal achievement and depersonalization, which are subdomains of burnout, are also related to ageism. Emotional exhaustion is the most important component of burnout, and in reaction to emotional exhaustion and demand, individuals seek to maintain a more distant or depersonalization attitude toward clients [43]. Nurses who harbor negative feelings and attitudes toward older people can treat the older patient unfairly due to prejudice and discrimination, denying them the opportunity to receive appropriate health and nursing care [44], which can be a factor for health inequality [45]. Therefore, it is important to confirm the extent and influencing factors of ageism and for nurses to recognize their own ageism in the hospital environment [27]. Moreover, management of nurse burnout is critical for increasing access to the health care and treatment needs of older patients, which continue to rapidly increasing in the current aging era, and for improving the quality of nursing. In particular, given that tertiary general hospitals have been known to accommodate a high number of older patients with severe disease conditions [34], policies that account for the characteristics of hospitals are required.

Additionally, the current study found that preference for geriatric nursing was a factor affecting nurse burnout. In particular, we found that that respondents who did not prefer geriatric nursing had increased ageism scores. Studies have shown that preference for geriatric nursing is a factor that predicts a positive attitude toward older patients [34] and that those who had less preference for geriatric nursing exhibited increased negative prejudice against an older patient [27]. This is presumed to be strongly associated with depersonalization [31], a sub-area of burnout that causes the subject to develop negative and cynical attitudes and emotions, which was also relatively high (a score of 3.06) among our study participants. Given that negative reactions to others can be a factor that increases emotional exhaustion, which is the most core aspect of burnout [31], preference for older patient care has ultimately been considered to affect burnout. Considering that preference for geriatric nursing is also a factor affecting ageism [27], research on personal and organizational factors that do not favor geriatric nursing are needed to break down psychological barriers to the older patient and establish policies that reflect the characteristics of geriatric nursing. In addition, we believe that geriatric nursing education is necessary to increase understanding and awareness of geriatric nursing.

Our results revealed that respondents who had experience in volunteer work related to older people had low scores on ageism. This finding is consistent with those presented in a study on ageism among college students, which showed that students with experience in volunteer work for older people had low levels of ageism [46]. Volunteer work is an activity that begins with voluntary motivation. In the process of volunteer work, positive relationships with older people can be formed, and in the process of helping older people, individuals can develop a sense of accomplishment or have a positive experience that can eliminate stereotypes or prejudices about older people [46]. Therefore, opportunities that promote interaction with older people and positive contact experiences should be encouraged [34]. In particular, a curriculum that allows undergraduate student nurses to accumulate positive experiences with older people should be established. Furthermore, as a care provider, human rights education should be strengthened so that nursing students have an attitude to advocate the human rights of older people. In addition, in order to continuously improve the caring attitude of nursing students for older people, it is necessary to prepare an educational strategy to understand the various characteristics of older people and acquire various knowledge necessary for geriatric nursing by providing sufficient opportunities to make practical contact and interact with the general older population before practicing geriatric nursing [34].

Our findings revealed that respondents with anxiety about aging had high ageism scores. This is consistent with previous research showing that anxiety about aging and ageism were positively correlated [47]. In a similar context, another study found that anxiety about aging was a factor affecting ageism [27]. Aging anxiety refers to worry or fear about getting older [34]. Nurses experience chronic disease or death while caring for older patients [48], which may alter their perception of aging [34]. In particular, one study showed that nurses at geriatric hospitals who care for older patients most closely develop a more negative perception of old age than do nurses working elsewhere [49], which may lead to anxiety about their own aging [34]. A high level of anxiety about aging among nurses has been strongly associated with negative and biased attitudes toward older patients [50]. This can be a factor that reduces the quality of older people care and increases ageism, which promotes nurse burnout. Therefore, continuous education is needed so that nurses can recognize various phenomena related to aging, such as chronic diseases and death, as a part of life and develop the right knowledge and attitude.

Korea has already begun to enter an aging society as evidenced by the significant increase in older population and the gradual rise in proportion of older patients visiting hospitals [34]. Nurses may develop negative perceptions and attitudes toward older patients depending on each individual’s experience while encountering several older patients, which can be a factor leading to increasing ageism and burnout among nurses. However, research on ageism and burnout among nurses is still lacking. The current study investigated levels of ageism among nurses at a tertiary general hospital with a high proportion of seriously ill older patients and identified its impact on nurse burnout, thereby establishing a basis for interventions that aim to reduce nurse burnout. This study confirmed that ageism was strongly associated with nurse burnout, highlighting the need to develop interventions that can alter nurses’ perception of older patients and promote emotional change. In addition, through intervention studies that increase nurses’ preference for geriatric nursing, we intend to improve the quality of geriatric nursing by reducing the ageism and burnout of nurses.

## Conclusion

As the utilization of healthcare services by older adults increases, nurses are becoming key agents in addressing health-related issues and promoting the well-being and quality of life among older patients. Nurses caring for older patients often experience high levels of physical and emotional exhaustion, which can lead to a loss of motivation, feelings of helplessness, and burnout. Unfortunately, limited domestic and international studies have been available on the relationship between ageism and nurse burnout.

To determine the extent of ageism among nurses experienced in caring for older patients, a structured questionnaire designed to collect information on general characteristics, ageism, and burnout were provided to the nurses.

Overall, our findings showed that ageism and each dimension of ageism were significantly correlated with burnout and each of dimension of burnout. This suggests that higher levels of ageism were significantly correlated with higher levels of burnout. Moreover, multiple regression analysis found that ageism (B = .1.066, p < .001), gender (female) (B = .530, p = .003), and preference for geriatric nursing (no preference) (B = .357, p = .002) significantly affected burnout, with such variables having an explanatory power of 40.4%. Ageism (β = .521) was found to have the greatest impact on burnout, followed by preference for geriatric nursing (β = .158) and gender (β = .082).

Nurses who harbor negative feelings and attitudes towards older people can treat the older patient unfairly due to prejudice and discrimination, denying them the opportunity to receive appropriate health and nursing care, which cause health inequalities. Therefore, it is important to confirming the extent and influencing factors of ageism among nurses and for nurses to recognize their own ageism in the hospital environment. This study confirmed that ageism was strongly associated with nurse burnout, highlighting the need to develop interventions that can alter nurses’ perception of older patients and trigger emotional change.

## Supporting information

S1 Dataset (XLSX)

S1 File (PDF)
