# Supplementary material for: Effects of ageism on burnout among clinical nurses
Source: PLoS One. 2024 Nov 7;19(11):e0313043. doi: 10.1371/journal.pone.0313043 (PMC11542876; doi:10.1371/journal.pone.0313043)
Supplement: S1 File — (PDF) [file pone.0313043.s002.pdf]

Version 1.0, 06/01/2023

All contents investigated in this survey are only for statistical purposes and are protected by the Statistical Law (Articles 33 and 34).

ID			

The Effect of ageism on burnout among nurses

Hello,

This survey is to investigate the impact of ageism towards the old people on burnout among ward nurses. Your sincere answers will be used as important data for understanding this issue and help establish systems to predict and prevent nurse burnout in clinical settings in the future, and will not be used for any other purpose.

You have the right not to participate in this survey and there will be no penalty for not participating in the study. You can also stop participating in the study by your own will, even while the study is in progress.

Respondents' data will be used only as a research purposes, and respondents' information is thoroughly protected in accordance with Article 33 of the Statistical Law, so we ask that you respond faithfully to all questions so that accurate data can be collected.

Thank you for participating in this study.

If you have any questions while filling out the questionnaire, please contact the principal investigator below.

May, 2023

▪ Principal investigator : Prof. Eunhee Shin, Department of Nursing sciences, Sangji University
(HP: 010-6278-9622)

Consent form

I fully understand the purpose of this study, and I understand and agree to the use of the data I have responded to. I also confirm that I have been informed that I can withdraw my consent at any time if I wish.

Agree □ Not agree □

Name: (Signature)

※ The next question is about **ageism.** Please put a √ in the box that applies to your opinion.

	Strongly disagree	Disagree	Agree	Strongly agree
1. I personally would not want to spend much time with an old person.				
2. I would prefer not to go to an old people's gathering, if invited.				
3. I would prefer not to live with an old person.				
4. It's a lot of fun to be with an old person.				
5. I don't like it when old people try to make conversation with me.				
6. Most old people are interesting and individualistic.				
7. I often avoid eye contact with old people.				
8. It is best for older people to live apart so as not to bother others.				
9. Most old people should not renew their driver's licenses.				
10. Seniors have virtually no need for local sports facilities.				
11. Older people deserve the same freedoms and rights as other members of our society.				
12. It's sad to hear about the plight of old people.				
13. Most older people live trapped in the past.				
14. When you're around older people, it's probably normal to feel depressed.				
15. Old people complain more than young people.				
16. Most older people are not interested in making new friends, but rather prefer to spend time with friends they have known for a long time.				
17. Most older people are happiest when they are with people of similar age.				
18. Most older people try to save up their money or possessions rather than giving them away.				

※ The next question is about psychological burnout. Please mark (√) the appropriate box according to your usual thinking

	None at all	Less than once a year	Less than once a month	a few times a month	Once a week	a few times a week	every day
1. I feel mentally exhausted from work.							
2. I'm exhausted at the end of a long day's work.							
3. I wake up in the morning and feel discouraged when I think about having to go to work again today.							
4. I can easily understand the subject's feelings.							
5. I feel like I'm treating some of my subjects like objects without personalities.							
6. Working with people all day is really hard for me.							
7. I deal effectively with the subject's problems.							
8. I am completely exhausted from my work.							
9. I feel like I am making a positive impact on other people's lives through my work.							
10. I've become more and more insensitive to people since I've been in this job.							
11. I'm afraid this will make me emotionally dry.							
12. I feel very energetic.							
13. I feel frustrated with my work.							
14. I feel like I'm working too hard.							
15. I don't really care what happens to any subject.							
16. It is very stressful for me to work while dealing with people directly.							
17. I can easily create a comfortable atmosphere for the subject.							
18. I feel very fulfilled after working closely with the subject.							
19. I have accomplished many valuable things through this work.							
20. There are times when I feel like I'm helpless.							
21. I deal with emotional problems very calmly while working.							
22. I feel that the subjects are blaming me for some of their problems.							

※ Here are some questions about your general characteristics. Please fill in each question.

1. Gender?

____① Male ____② Female

2. Age? ______________year

3. Which of the following is your educational background?

____① College ____② University ____③ Master's degree or higher

4. What is your marital status?

____① Single ____② Married

5. Do you have a religion?

____① Yes ____② No

6. What is your working period at the current workplace? ____________ month

7. Which type of healthcare institution do you currently work at?

____① Tertiary hospital ____② General or specialized hospital

____③ Long-term care hospital ____④ Other ____________________

8. Which department are you currently working in?

____① Internal ward ____② Surgical ward

____③ Intensive care unit(Internal/Surgical) ____④ Outpatient

____⑤ Emergency room ____⑥ Others ____________________

9. What type of area did you live in while growing up?

____① metropolitan areas ____② Local city

____③ Rural area ____④ Others ( )

10. Have you received any educational related to old patients?

____① Yes ____② No

11. Have you ever lived with an old person in the past?

____① Yes ____② No

12. Are you currently living with an old person?

____① Yes ____② No

13. Have you ever participated in volunteer work related to old people?

____① Yes ____② No

14. Have you ever felt anxious about aging?

____① Yes ____② No

15. Which of the following are your preferences for nursing care for older patients?

____① Very preferred ____② Preferred

____③ Average ____④ Not preferred

____⑤ Not very preferred

♣ Thank you very much for your response!! ♣
